# “On paper it was a surgical success, but the reality was very different”: A qualitative interview study using narrated casuistry to explore patient context in specialist care

**DOI:** 10.1371/journal.pone.0339353

**Published:** 2026-01-12

**Authors:** Linda Modderkolk, Yvonne Schoon, Hugo Touw, Eva Stortelder, Marieke Voet, Yvonne Engels, Anne B. Wichmann

**Affiliations:** 1 Department of Anaesthesiology, Pain and Palliative Medicine, Radboudumc, Nijmegen, the Netherlands; 2 Department of Geriatrics, Radboudumc Center for Integrated Care, Radboudumc, Nijmegen, the Netherlands; 3 Department of Intensive Care Medicine, Radboudumc, Nijmegen, the Netherlands; 4 Department of Global Public Health and Bioethics, Julius Center for Health Sciences and Primary Care, University Medical Center Utrecht, the Netherlands; 5 Department of Pediatric Surgery, Queen Fabiola Children’s hospital, University of Brussels, Belgium; Alexandria University Faculty of Nursing, EGYPT

## Abstract

**Objective:**

Approaches such as shared decision-making emphasize effective patient-provider communication to ensure that patient preferences are integrated into clinical practice. However, they often overlook crucial aspects of patient context, which can affect the appropriateness and feasibility of care planning. Applying a contextual care model addresses this gap, yet the extent to which relevant patient context is considered in routine clinical practice remains largely unknown. Previous studies have primarily focused on the United States, leaving a gap in understanding how care is contextualized in Europe. This study explored how medical specialists in the Netherlands interpret, perceive and incorporate patient context into care planning.

**Methods:**

A qualitative interview study was performed with 18 medical specialists from 11 specialties working in academic and peripheral hospitals. Open coding was applied to explore how specialists defined and perceived patient context. Directed content analysis, based on the 4C coding process, was applied to analyze narrated case examples of contextualized and non-contextualized care.

**Results:**

All participants acknowledged the importance of patient context for providing appropriate care, though they differed in how broadly they defined it – from physical and cognitive abilities, to social, emotional and environmental circumstances. Context was considered most relevant for making treatment decisions and, by half of the participants, for ongoing monitoring of treatment. Participants also described cases where contextualization developed over time or was shared among team members. Key challenges included distinguishing context from patient preferences and sufficiently probing red flags to inform care planning.

**Conclusion:**

Medical specialists emphasized the essential role of patient context in enabling personalized and efficient decision-making, enhancing care monitoring, and ultimately improving clinical outcomes. However, systematic and continuous integration of contextual information into decision-making remains challenging. Addressing this requires proactive exploration, improved communication training, and organizational support to embed contextualized care in routine practice.

## Introduction

In recent years, healthcare has advanced rapidly with technological innovations such as precision medicine, alongside a growing emphasis on person-centered care that prioritizes patients’ multidimensional needs and preferences [1]. Both developments rely on effective patient-provider communication to ensure appropriate treatment decisions. Shared decision-making (SDM) is a process increasingly adopted by physicians to integrate patient values, goals, and preferences into care planning [2]. While SDM aims to elicit patient preferences, it often fails to account for the broader context of the individual patient, including social, economic, cultural, and environmental factors that may shape care decisions and outcomes [3]. Consequently, physicians may lack sufficient awareness, knowledge, and communication skills to effectively identify, discuss, and integrate this relevant context into care planning [4,5].

**Fig 1:**
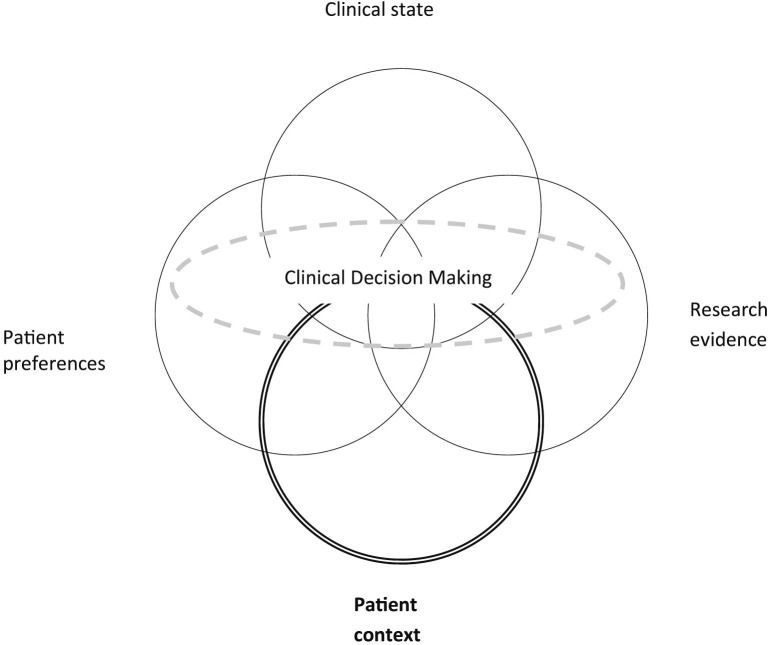
Clinical decision-making according to the contextual care model [4].

The contextual care model addresses the gap between evidence-based medicine (EBM) and person-centered care by explicitly incorporating patient context into clinical decision-making. It introduces patient context as a fourth component to the existing clinical decision-making framework, which traditionally comprises three elements: the patient’s clinical condition, research evidence, and patient preferences (see Fig 1). By considering patient context as an additional dimensions, the model aims to enhance the planning and delivery of truly person-centered care [4].

Patient context is defined as “all that is expressed outside of the boundaries of a patient’s skin that is relevant for planning the patient’s care” [6]. The model encourages clinicians to recognize, explore and integrate relevant contextual factors—such as access to care, living environment, and emotional state—into treatment planning [7]. Continuously considering potentially relevant patient context in every encounter helps reduce the risk of contextual errors, a specific type of medical error that often leads to inappropriate treatment and costly complications [5]. Evidence indicates that contextualizing care improves patient outcomes and reduces costs, as demonstrated by a 2.5% reduction in hospitalizations and $25 million in savings when care contextualization increased [8]. Moreover, contextualizing care does not lengthen consultations [5].

Most empirical evidence on contextualizing care originates from studies conducted in the United States and primarily focuses on internal-medicine or primary-care settings using standardized patients and patient-collected audio recordings. Evidence on how medical specialists across hospital-based disciplines interpret and systematically apply patient context in routine practice – particularly outside the U.S. - remains limited. Although there is a growing emphasis on a multidimensional view of health in the Netherlands, a Dutch survey study revealed that physicians still assess health narrowly and biomedically [9]. Despite efforts to broaden physicians’ attention to multidimensional care provision and to improve their communication skills to elicit patients’ preferences [10,11], medical education and existing guidelines still provide little attention to contextualizing care [12]. However, it is unclear whether this also applies to current clinical practice. Therefore, this study aimed to investigate I) how medical specialists in the Netherlands interpret contextualizing care and how they perceive its relevance, and II) how they self-report their current practice of contextualizing care.

## Methods

### Design

The primary aim of this interview study was to explore how medical specialists in Dutch hospitals interpret, perceive and practice contextualizing care. Therefore, the interview consisted of questions exploring these themes and collecting casuistry illustrating the relevance of patient context for care planning. The Consolidated Criteria for Reporting Qualitative Research (COREQ) checklist was used to report this study and its findings [13] and can be found in Supplement 1 (S1 File).

### Theoretical framework

**Fig 2:**
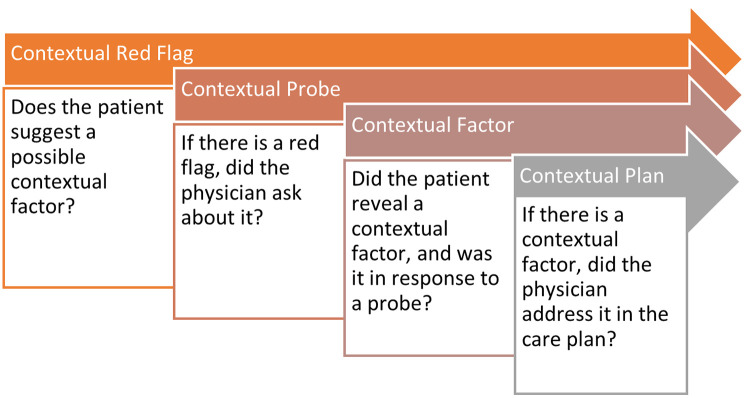
The 4C coding process adopted from Weiner [5].

The theoretical framework for this study was inspired by the contextual care model as developed in the U.S. [5]. In this model, patient context is categorized into twelve distinct domains, which are further grouped into two primary categories: patient circumstances and patient behaviors [7]. See Table 1 for definitions for the contextual domains. The casuistry collected in the interviews was analyzed using direct content analysis informed by the Content Coding for Contextualization of Care (4C) process [7]. The 4C coding process is traditionally used to analyze contextualization of care in consultations and also encompasses information from the medical record. We innovatively applied it to analyze narrated cases of consultations. The 4C coding process involves: 1) recognizing a contextual red flag, 2) contextual probing of the red flag, 3) deciding if it reveals a relevant contextual factor, and if so, 4) integrating the identified factor into a contextualized care plan. Fig 2 shows a visual representation of this process.

**Table 1:** Definitions for the contextual domains [7].

	Contextual domain	Definition
**Circumstances**		
1.	Access to care	The patient’s ability to receive care in a timely manner.
2.	Competing responsibility	An obligation or commitment the patient has that impacts their ability to manage their health care.
3.	Social support	The patient’s access to a supportive network of individual(s) able to assist if needed.
4.	Financial situation	The patient’s ability to afford health and health care needs.
5.	Environment	The physical and social setting that encompasses a patient.
6.	Resources	The possessions and materials available to a patient that can facilitate a person’s ability to manage their care.
**Behaviors**		
7.	Skills, abilities and knowledge	A patient’s intellectual understanding and physical ability to manage health care.
8.	Emotional state	The emotional condition of a patient as it relates to their ability to manage their health care.
9.	Cultural perspective/spiritual beliefs	The customs or a faith-based practice a patient has that impacts health care.
10.	Attitude towards illness	The feelings a patient has towards their condition that impacts their ability to manage it.
11.	Attitude towards health care provider and system	The patient’s feelings and attitudes towards their providers and the health care system that impact their ability to manage their health care.
12.	Health behavior	The patient’s actions and lifestyle choices that impact their health care.

### Participants and data collection

Using purposive sampling, we selected and interviewed medical specialists working in a hospital in the Netherlands, between June 1^st^ 2021 and February 28^th^ 2022. A variety of participants was included in terms of medical specialty, hospital type (peripheral versus academic hospital), age, work experience, and gender identity. Medical specialists were approached by email using the researchers network and a snowball approach. They received an information letter about the aim of the study, including the three main questions (see paragraph below) in the interview. A few examples were given to illustrate the definition of patient context. Considering the latest insights on data saturation and information power in qualitative research, we selected a sample of 18 specialists. The combination of uniformity in profession and diversity in specialty, gender, age, and work experience provides a strong foundation for a rich and nuanced description of the insights obtained [14–16].

The interview guide consisted of three open-ended questions and was followed by sub-questions to specify the answers. (See S2 File) The three main questions explored 1) interpretation and perceived relevance of patient context for clinical decision-making according to participants, 2) one or more cases in which participants integrated relevant patient context into care planning and 3) one or more cases in which participants (unconsciously) refrained from integrating relevant patient context into care planning. The interviews were conducted via video calls and lasted between 30–60 minutes. All interviews were conducted in Dutch by the same researcher (LM) who had no prior acquaintance with the participants. For the first two interviews, a second researcher (AW) attended the interview to refine the guide. After obtaining oral consent, the interviews were recorded and transcribed verbatim. Transcribed verbatims were send to the participants for consent and correction.

### Data analysis

The interpretation and perceived relevance of contextualizing care was analyzed using conventional content analysis. The codebook was developed inductively. The described casuistry was analyzed using a directed content analysis approach informed by the 4C coding process, and the codebook was developed according to these elements [17]. Since we used casuistry described by participants, probes were difficult to recall. According to ‘Simon’s Rule’, we considered a probe valid when the context of the story implied its presence, although the respondent did not directly mention it: ‘If the health care provider makes a statement (rather than asking an overt question) that demonstrates awareness of the red flag and the patient responds by revealing a contextual problem relating to the red flag, the health care provider would be given credit for a contextual probe.’ [7] Two researchers (LM and AW) coded the first half of the interviews line-by-line. Once agreement was reached on the codebook, the other half of the interviews were coded by one researcher (LM). Categories were defined in two peer sessions (LM, AW, YE). Also, an affinity diagram was created, which guided the description of the results. Analysis software ATLAS.ti 9.1.6. was used.

### Ethics approval

Since participants were not subject to treatment or required to behave in a certain way, the Medical Review Ethics Committee region Arnhem-Nijmegen granted an exemption from further assessment of the submitted protocol for this study (case number 2021–8159) considering the Medical Research Involving Human Subjects Act. The conduct of this study was therefore approved, provided that other relevant legislation such as the GDPR and the Declaration of Helsinki was complied with. Oral informed consent was obtained and recorded from all participants at the beginning of the interview. The anonymity of the participants and the patients in the discussed clinical cases was guaranteed by removing all personal retraceable information from the transcripts.

### Research team and reflexivity

The research team consisted of six women (LM, YS, ES, MV, YE, ABW) and one man (HT), with backgrounds in anesthesiology, geriatrics, intensive care, surgery, counseling, medical ethics, and meaningful healthcare. Together, the team spans clinical practice, research, and education across multiple institutions. We acknowledge the potential for positionality and confirmation bias, as our backgrounds may shape how we identify, interpret and prioritize aspects of contextualized care. To mitigate this, we engaged in reflexive discussions to challenge our assumptions throughout the research process, sought diverse perspectives through the casuistry and integrated discussion and literature on health disparities.

## Results

A total of 18 medical specialists (12 women, 6 men) took part in the study. 12 participants worked in an academic hospital and 6 in a peripheral hospital, representing 11 different specialties. Participants’ characteristics are described in Table 2. For each research question, the findings are illustrated with quotes and two exemplary cases of achieved and missed integration of patient context in consultations and care planning showing all 4C elements (see Box 1 and Box 2).

**Table 2:** Participants’ characteristics.

	Overall (n = 18)	Women (n = 12)	Men (n = 6)
**Age**			
30-39 years	1	0	1
40-49 years	8	8	0
50-59 years	5	3	2
60 + years	4	1	3
**Specialty**			
Anesthesiologist	1	1	0
Cardiologist	1	1	0
Intensivist	2	1	1
Pediatric intensivist	1	1	0
Surgeon	4	2	2
Palliative care physician	1	1	0
Oncologist	1	1	0
Geriatrician	4	3	1
Pulmonologist	1	1	0
Neurologist	1	0	1
Nephrologist	1	0	1
**Work setting**			
Peripheral hospital	6	5	1
Academic hospital	12	7	5

### I. The interpretation and perceived relevance of patient context for medical care

All participants had a similar understanding of patient context, which included a patient’s physical and cognitive abilities, daily responsibilities (such as work or caregiving), personal interests or hobbies, and the strength and structure of their social network. However, they differed in where they placed their emphasis. Some primarily focused on physical capabilities when assessing a patient’s ability to undergo treatment, while others also considered factors like living conditions and social circumstances. There seemed to be no significant differences in interpretation and perception of the relevance of patient context across the demographic characteristics of participants. However, some mentioned working experience to be an important factor in their ability to address patient context in daily practice, as this participant describes:

*“To become a good doctor, you first need to learn how to excel in the medical-technical aspects, and that requires an enormous amount of focus and energy. As a result, there’s little room to pay attention to the surrounding context at that stage. However, it’s important to develop that awareness as well.”* (R1)

All participants acknowledged the importance of attentively considering relevant patient context when providing medical care. Many emphasized that this attentiveness is a fundamental aspect of a medical specialist’s professional attitude. While all participants underscored the importance of patient context, their interpretations of its scope varied. Some participants focused on pragmatic considerations, emphasizing how contextual factors shape treatment logistics or adherence *(‘By understanding factors earlier, you can improve treatment quality and work more efficiently as a caregiver.’* (R14). Others highlighted the direct causal link between patient context and health outcomes, positioning context as an intrinsic determinant of disease *(‘I see elderly, single women struggling financially, saying they can’t afford heating or healthy food. This leads to high stress, poor nutrition, and constant worry—major risk factors. Telling them to try yoga or relax isn’t helpful when they lack the resources to address their basic needs.’* (R18).

Overall, participants highlighted that having ears and eyes for relevant patient context is particularly valuable at two critical stages. Firstly, a majority of participants (14 out of 18) considered patient context to be relevant when making medical decisions. Aligning treatment decisions with patient context was considered important to improve care quality and managing (potential) complications or complex cases. Interestingly, some examples given to illustrate this point, (11 out of 45 cases, by 8 different participants) demonstrated patient preferences rather than patient context, for example in this case:

*“Last week I saw a patient in the preoperative clinic that I found quite interesting. She was a woman, not yet 70, with breast cancer, scheduled for breast surgery. She was actually quite fit, and from an anesthesiology perspective, a breast surgery is a minor, very safe procedure—nothing particularly complicated in my view. However, this woman was very clear: she did not want to be resuscitated under any circumstances. She even had a DNR medallion and had clearly thought it through carefully—she didn’t want any life-prolonging measures.”* (R1)

Although preferences were deemed essential for determining the right treatment plan in these cases, they lacked the relevant contextual information that shaped them. Being aware of relevant patient context, as observed across other cases, often enhanced participants’ understanding of patient preferences and their ability to incorporate these into care planning, as illustrated by this participant in her geriatric assessment of a patient:

*“I saw a woman and her husband during my preoperative consultation for the head and neck clinic. She had a tumor on the inside of her lip, and while further tests were being conducted that day, she firmly declared, despite prior consultations with other doctors, “I won’t do anything about it.” The required surgery was extensive and would likely impair her ability to speak temporarily. She also understood that it would leave a significant impact on her facial appearance. Through my assessment, I learned she had worked in the fashion industry. She and her husband, who had no children, described their life as wonderful, filled with travel and meaningful experiences. She told me, “Two things are very important to me: being able to communicate clearly and looking presentable.” She was well-groomed, cognitively sharp, and deeply aware of the implications of the surgery. For her, the temporary loss of speech and the disfiguring effects of the procedure were unacceptable. She said, “That’s why I won’t undergo the treatment.” She explained her decision further, reflecting on their lives and experiences. Both had lost their parents relatively young to illness, but they felt fortunate to have lived over 80 years with few hardships. She concluded, “We’ve had a beautiful life, and I don’t want to live disfigured or unable to speak properly.” In the end, I can tell you that she completed all the additional tests as planned and even participated in the multidisciplinary team discussion. The recommendation was surgery followed by radiation therapy. On Friday, she had another meeting with her primary treating physician. Despite knowing that declining treatment meant she would likely pass away within six months due to the rapidly progressing tumor, she stood firm in her decision. Fully aware of the consequences, she made her choice with remarkable clarity and determination.”* (R15)

Occasionally, a participant perceived context to become more significant as the severity of the condition increased. Similarly, participants generally regarded context as less relevant in younger patients and/or straightforward cases, such as performing a tonsillectomy on a healthy 25-year-old.

Secondly, patient context was considered essential in continuously monitoring how various aspects of patient context affected the treatment process *after it started* by half of the participants (9 out of 18). This quote highlights the impact of relevant patient context on a patient’s treatment process:

*“When patients undergo dialysis treatment, they visit the hospital three times a week for several hours each time. This routine provides them with a structured framework, including frequent contacts with healthcare professionals. […] However, after a kidney transplant, patients initially require weekly check-ups, which later decrease to once every two or three weeks. This shift often removes the structured support system they relied on during dialysis. We’ve encountered cases where this transition revealed significant issues at home. Some patients struggled to care for themselves, lacked transportation to attend appointments and sometimes had no money to cover travel costs. In other instances, we discovered patients living in unhygienic environments while on immunosuppressive medications, which increased their vulnerability to infections. These situations highlight the gaps in post-transplant care and the critical need for ongoing support.”* (R14)

The relationship between participants and their patients was often described as beneficial to eliciting and monitoring relevant patient context, especially in longer treatment processes. However, a noted risk was the tendency for assumptions to develop over time in long-term patient-provider relationships, which could prevent providers from routinely verifying patients’ previous statements, as this quote illustrates:

*“I had a patient, a 63-year-old woman with metastatic breast cancer. After the cancer spread to her brain, she became very ill and underwent surgery and radiation, with the plan to resume chemotherapy once she recovered. Initially, she agreed, but later kept postponing, saying she wasn’t well enough. When a case manager suggested an in-person meeting, she finally admitted*, ‘I don’t want chemo anymore’. *I’ve known her for a long time, and she was always very clear—she did everything, always wanted every possible treatment, even more rather than less. It was the first time she asserted herself after always following medical advice, and I realized I had never directly asked her if she still wanted to continue treatment.”* (R7)

Contextual factors were recognized to impact the treatment process in three main ways: 1) by directly affecting the patient’s physical condition (e.g., in cases of stress-related heart disease), 2) by affecting the patient’s ability to undergo a medical treatment (e.g., a patient’s emotional state as described in Box 1), and 3) by affecting the treatment process (e.g., a patient without transportation facilities to the hospital).

Although all participants recognized the importance of contextualizing care, several cited instances where colleagues failed to integrate context into care planning. Crucial contextual conversations, particularly those on advance care planning (ACP), occurred too late or not at all, resulting in compromised care and diminished quality of life. They stated that this lack of timely conversation also affects the participants’ own care delivery, impacting their practice and ability to provide optimal patient care.

Furthermore, some participants highlighted the broader impact of systemic factors, such as poverty, unequal opportunities, and healthcare financial structures, on patient context and medical care. Even though they try to address it on the patient level whenever possible, they also experienced those factors to be beyond their ability to solve. At times, this negatively impacted their motivation as it created a sense of frustration, as expressed by this participant:

*“For example, a child with very severe asthma, or untreated asthma, is often from a more disadvantaged social background. We sometimes encounter children with extremely poor dental health, who are given bottles of cola or Fanta to drink, and who will only eat McDonald’s — and their parents provide it. […] We inevitably form opinions about such situations. For instance, when parents smoke heavily while their child suffers from asthma, there is little you can do to change that dynamic. You can explain that drinking cola is harmful to the child, but changing that behavior is unlikely. I’ve come to accept that this is something you cannot easily fix. You can address it, share your perspective, and raise awareness, but it won’t make much of a difference in practice.”* (R5)

### II. Contextualizing care in practice

#### Overview.

In the interviews, participants described cases in which context was integrated (*25 contextualized cases*) and cases where they recognized – in hindsight – context had been overlooked *(9 non-contextualized cases*)*.* These cases were analyzed and described below, inspired by the 4C coding process (see Fig 2) to determine how care is being contextualized in practice. An overview of the identified 4C elements can be found in Supplement 3 (S3 File). Two integral cases have been included for illustration of our findings: a contextualized case (Box 1) and a non-contextualized case (Box 2).

### 1. Contextual red flags

#### Contextualized cases.

In almost all described contextualized cases, participants identified a red flag (96% of the cases). These red flags were detected through verbal communication, non-verbal cues from the patient, or medical facts. Verbal communication was the most frequently cited source of red flags (72% of the cases), with a patient’s resistance to treatment options being the most recognized verbal indicator (44% of the verbal red flags). Regarding non-verbal cues (12% of the cases), participants recognized the patient’s physical and cognitive abilities, as well as their emotions as red flags. Under medical facts (44% of the cases), red flags included biomedical aspects, such as the patient’s physical condition and frequent hospitalizations, or patient behaviors like arriving late or missing appointments. In some cases, multiple red flags were identified, and their combination heightened the likelihood of participants recognizing the need for further exploration, as demonstrated in this example from a palliative care setting:

*“At the request of the general practitioner, who often asks if we do home visits because he knows their added value, I visited a man. He was in his room, and pain was a major issue. We couldn’t get it under control—his morphine pump was set extremely high, and there was a lot of trouble around it. We were really wondering: a) why isn’t it working? and b) what can we do next? However, he didn’t want to take many of the other medications we proposed. We had several other options, including injections, but he refused them all. I found myself thinking, “Well, on the one hand, you’re complaining about the pain, but on the other hand, you’re rejecting the solutions we have to offer.”* (R4)

#### Non-contextualized cases.

In non-contextualized cases where red flags were missed (100% of the cases), the same three main categories emerged in hindsight: verbal communication, non-verbal cues, and medical facts. Upon reflection, participants noted missed red flags in verbal communication, such as resistance to treatment options, unknown treatment preferences, and in one instance, a patient’s complex social situation. Non-verbal red flags that were overlooked included the patient’s physical and cognitive capabilities and emotions. Missed red flags were evenly spread across the categories.

### 2. Contextual probes

#### Contextualized cases.

Probes were identified in 68% of the described contextualized cases. Participants mainly probed red flags to understand the reason behind them (e.g., to understand why a patient refused a catheter), to explore underlying reasons for patients’ treatment preferences (such as asking about the level of acceptable suffering from potential side effects related to meaningful hobbies), and to uncover potential implications for treatment decisions, as illustrated by this surgeon:

*“Recently, we had a patient who had broken his elbow and upper arm. It was a complicated fracture, but the alignment was still fairly decent. The procedure to fix it surgically would be quite complex, and the patient was an older man with multiple comorbidities. He had already had a prosthesis and dealt with infections, making him far from an ideal candidate for surgery. My colleague had already said, “We shouldn’t operate; there’s a good chance it will heal well on its own.” However, I happened to see the patient, and he came in with a cane, saying, “I have no function in my other hand because of other problems.” This meant it was his only working hand. I then had a renewed discussion with the patient, asking, “What exactly are the risks, and is the risk worth it to ensure you regain better function in your left arm—the only arm you really have”* (R13)

Although the majority of participants indicated to have probed the reported red flags in the contextualized cases, the quality of these probes varied. In some cases, the red flag was acknowledged and followed up with a probe that focused on exploring the consequences without delving into the meaning behind the red flag. For example, when a participant recognized and explored a red flag, such as a patient’s wish to keep walking her dog after surgery, the underlying reason for this wish remained unclear. This lack of deeper exploration makes it difficult to ascertain whether the appropriate contextual factor was effectively addressed.

In addition to *what* participants probed, we also analyzed *how* they conducted their probing. In most instances where participants probed the red flag, it happened during the same conversation with the patient (53% of the cases). Respondents could also revisit the issue during a follow-up appointment or repeatedly over time. Sometimes, the task of probing was delegated to another healthcare professional, such as the general practitioner (GP), or carried out collaboratively with another professional, like a GP. In a few cases, questions were directed to a colleague, such as the GP or another involved healthcare professional, instead of the patient. In one case, the questions were part of a structured patient history-taking process.

#### Non-contextualized cases.

In the non-contextualized cases, participants only discovered the existence of relevant patient context in hindsight. Probing therefore did not take place. Of all non-contextualized cases described during the interviews, in just one case a contextual probe was identified (9% of the cases). In this instance, the participant recognized the red flag (e.g., the patient’s angry behavior) and attempted to probe it. However, the patient did not respond honestly, which prevented the discovery of the underlying contextual factor – a psychiatric disorder. This information was eventually disclosed by the GP after the patient had terminated the treatment relationship with the participant. Several participants reflected on their probing, or the lack thereof, in the non-contextualized cases:

*“And then I think: should I have asked more questions? In hindsight, did I do it well enough? Did I really ask him clearly enough what he was currently capable of and what he wanted from the surgery? Did I properly discuss the option of doing nothing with him and his family?”* (R8)

### 3. Contextual domains

#### Contextualized cases.

In 96% of the cases, a contextual factor was identified, within seven different contextual domains. Five of these align with domains from the contextual care model (see Table 1). Two domains, ‘meaning’ and ‘sexuality’, were added to the model during the analysis. ‘Sexuality’ involved intimacy concerns that influenced patients’ behavior and choices, for example, a patient who refused a catheter to preserve his ability to maintain sexual intimacy with his wife. ‘Meaning’ referred to how patients navigated their illness within the broader context of their lives, as illustrated by the case of the patient with a lip tumor in Part I (R15). The most frequently mentioned domain was emotional state (28% of the cases). This is defined in the model as ‘*the emotional condition of a patient as it relates to their ability to manage their health care*.’, and illustrated by this quote:

“*About 15 years ago, I first met a woman in her early forties who had been referred to me by another cardiologist, who found her case difficult. In another hospital, they had diagnosed her with poor heart muscle function during a time when she was struggling with various problems. However, when I reassessed her heart, I found the function was actually quite good, and she told me she was feeling much better emotionally at that point. As we talked more, she revealed a history of neglect in her youth—being ignored, unloved, and made to feel worthless. She shared that during periods when these memories resurfaced, her physical and emotional health would decline significantly. Later, while she was under my care, she experienced similar episodes, and during those times, her heart function visibly worsened on echocardiograms. This connection between her emotional state and heart function was invaluable for understanding her condition. It also strengthened our relationship, as knowing her history allowed me to better support and treat her during these challenging times.*” (R18)

Other frequently identified domains included skills, abilities, and knowledge (19% of the cases; e.g., a patient who struggles to understand how to take their medications), health behavior (19% of the cases; e.g., a patient’s preference for using weed oil instead of chemotherapy), and environment (19% of the cases; e.g., a child with severe asthma living with parents who smoke). No examples were given of the domains access to care, competing responsibility, social support, financial situation, resources, cultural perspectives/spiritual beliefs and attitude towards health care provider and system.

#### Non-contextualized cases.

In the non-contextualized cases, a contextual factor was identified in retrospect in 100% of the cases. The domains that were overlooked as recalled by participants were similar to those that were recognized, with the addition of the domain social support. No single domain stood out in the non-contextualized cases. In most cases, overlooking relevant context hindered a deeper understanding of the patient’s situation and preferences. Participants noted that this also led to missed opportunities to clarify or anticipate the treatment trajectory, ultimately resulting in suboptimal care. One participant illustrated this with the following case representing the domain *attitude towards illness*:

*“I was on call, and this woman was scheduled for emergency surgery due to an acute inflammation, which I was to perform as the on-duty physician. I had spoken to her briefly beforehand. The conversation was quick and somewhat superficial because it was clear what needed to be done. During the surgery, however, I encountered not one but three inflammations and treated all three. To my surprise, she was furious with me for doing so. I thought, as a conscientious caregiver, addressing additional findings seemed like the best course of action. If I had known her better, I would have better understood why, in this case, I should have simply gone along with her wishes. She said, ‘This is what needs to be done now because this is what really bothers me. The other issue has been present for a long time, and every treatment for it causes me immense discomfort, hassle, and distress—I don’t want that.’ You can debate how medically justified her stance was, but that wasn’t the point for her. That was the first thing. The second thing, which I only realized later, was that this was also about autonomy. She wanted to decide for herself whether or not to proceed. As I talked with her, it became clear that autonomy was incredibly important to her because she already had so many things in her life that she cannot control. That’s another layer of context.”* (R10)

### 4. Contextualized plans

#### Contextualized cases.

In 72% of the cases described, we could identify how participants contextualized treatment plans, in the remaining cases the exact contextualized plan remained unclear. Contextualizing plans often involved refraining from medical interventions, adjusting the treatment plan, or ensuring the right care after treatment completion. In other contextualized cases, solutions were arranged to address the identified contextual factors in different ways while continuing standard treatment, such as involving additional professional support during treatment.

Participants mentioned that the treatment plan was often discussed with the patient in a SDM process. However, there were instances where patients were unable to make decisions because they were either unconscious or cognitively impaired. In these cases, the responsibility of discussing treatment choices fell on their relatives or caregivers from another care institution. The following quote from a geriatrician illustrates the sometimes complex, intertwining components of a patient’s context and the impact on their treatment plan:

“*I once saw a lady who, in an impulsive act at home, had hit herself on the head with a hammer, leaving a literal hole in her skull. She was restless and confused, so I was called in and could help with medication, while the surgeons, who had OR space, wanted to operate immediately since the brain was exposed and there was a risk of infection. I stopped that because she could not give consent and her family refused to come as they were angry with her. So I took her to my ward where, once she woke up, we saw she was cognitively intact though functionally dependent, living in a shed without facilities and having become incontinent, and she explained she had not wanted to go to a nursing home and acted out of despair. Eventually I convinced the family to come, and when she woke she told them, “I was a bit stupid,” and things were forgiven. And in the end she did move to a nursing home where she blossomed with care and attention, the wound healed with antibiotics and granulation without infection, and she herself said, “I’m so old, it doesn’t matter,”. This showed me how important it is to take time, not rush to the OR just because there’s space, but to step back and look at what is really going on.*” (R15)

#### Non-contextualized cases.

In the non-contextualized cases, participants found that discussions about the patient’s context and associated treatment preferences were insufficient. Looking back, they felt that more in-depth discussions would have been necessary. They mentioned several examples of negative outcomes resulting from not contextualizing a patient’s treatment plan. These included treatment choices that did not align with patient context, unforeseen complications, limitations on a participants ability to make well-informed decisions during surgery and psychiatric admission of a patient due to sensitivity to psychopharmaceuticals (see the case in Box 2). In most cases, the participants questioned whether they should have taken different actions, recognizing that they will never know for certain if it would have made a difference.

**Box 1. An example of a contextualized case.**

**Participant:** “It was a relatively young man who made a very deliberate decision to undergo surgery and get a stoma, rather than attempting radiation or chemotherapy to shrink the tumor and hoping it would disappear with follow-ups and further treatments. I [the medical specialist] was somewhat surprised, as young patients often want to try everything—extra chemo, extra radiotherapy—despite the long-term consequences being hard to predict. Many would prefer to avoid a stoma or surgery if possible, but he chose otherwise. (*Contextual red flag*) He explained that his 16-year-old son had died in a car accident a few months earlier, and the family was still mourning. He told me, *‘I just want it gone. Cut it out, give me a stoma, and then I’m done. I don’t want to come back to the hospital, I don’t want any checkups, and I don’t want pre-treatment.*’ His wife asked, ‘*Are you sure about the stoma? It will be permanent*.’ But he replied, *‘If I have to keep doing checkups and face all this uncertainty, I can’t move on. I want to end this chapter so I can focus on grieving.*’ (*Contextual probe and contextual factor*) **Interviewer:** ‘And did it go as planned?’ **Participant:** ‘Yes [he got the surgery and stoma], he was an exceptionally satisfied patient. He even sent a card thanking us, saying he was pleased it was handled all at once and that he could now move forward.” (*Contextual plan*) *(R8).*

**Box 2. An example of a non-contextualized case.**

**Participant:** “I recall a man in his 70s who was seen by our geriatrics team preoperatively for the replacement of an aortic aneurysm. The surgery itself was performed, and he spent an extended six weeks in the ICU, followed by time on the ward before being discharged. On paper, this was considered a surgical success—there were complications, but he made it through. However, the reality was very different. The man was admitted to a psychiatric hospital shortly after discharge, and six months later, when I checked his file, he was still there. In hindsight, we missed several signs during the outpatient phase. If we had looked more closely, we would have noticed he was already on psychopharmaceuticals and had a prior psychiatric admission [*But was currently stable.*]. (*Missed contextual red flag*) Unfortunately, these details were neither asked about nor investigated. (*Missed contextual probe*) While we assessed his physical resilience, we failed to account for his psychological resilience, which was inadequate for coping with such a prolonged ICU stay, severe complications, and the difficult recovery process. (*Missed contextual factor*) The emotional toll led him into depression. When I reviewed his case later, he was still admitted to the psychiatric hospital, unable to recover from what had happened.” (*Missed contextual plan*) *(R15).*

## Discussion

This study shows that medical specialists in the Netherlands broadly acknowledge the importance of patient context for care planning but struggle to integrate it systematically into clinical practice. While all participants considered initial contextual communication essential for appropriate medical decision-making, about half stressed the importance of maintaining continuous contextual communication throughout the treatment process. The cases shared by participants illustrated both successful and missed contextualization, showing how patient context can shape clinical decisions. Five domains of the contextual care model were confirmed while two new domains – meaning and sexuality – emerged, highlighting areas often overlooked in clinical practice. The main challenges identified were distinguishing context from patient preferences and adequately probing contextual red flags.

### Interpreting the relevance of patient context

Participants described more contextualized than non-contextualized cases, which likely reflects how difficult it is to recognize missed context in hindsight and how easily biomedical issues or patient preferences can be mistaken for contextual factors. This tendency echoes what Foucault described as the medical gaze, where the clinical focus on the body can overshadow a patient’s personal and social circumstances [18]. Proactively exploring contextual factors – through structured processes such as SDM, Advance Care Planning (ACP) or Personal Perspective Elicitation (PPE) – could help clinicians broaden their view of the patient’s situation [3].

Earlier studies have shown that clinicians often overestimate their SDM abilities, while both preferences and contextual factors are rarely fully integrated into care planning [3]. This also raises a fundamental question: who is responsible for bringing relevant context into clinical encounters, the professional or the patient? Patients may not always realize which aspects of their lives are relevant to their treatment, therefore relying solely on a signal-driven approach is unlikely to be sufficient [19]. Our findings therefore suggest that combining proactive exploration with responsive follow-up seems more effective in identifying relevant context.

Half of the participants (9 out of 18) highlighted the importance of continuously monitoring patient context, in line with research linking ongoing contextualization to better communication and adaptation over time [2,6,20]. Although some participants felt that context mattered less in routine cases, even those straightforward treatments can be complicated by unexpected personal circumstances [21]. For example, an elective knee surgery may seem routine but becomes complex if the patient is a primary caregiver. Integrating brief contextual prompts into regular workflows could help anticipate and prevent such issues [22,23]. System-level interventions, such as Electronic Health Record (EHR) clinical decision support and contextual alerts, can help reduce errors. Still, verbal discussions remain essential for effectively integrating contextual insights into care [24].

At the same time, our findings should be interpreted against the backdrop of mixed evidence on the impact of contextualizing care. While studies in the U.S. have demonstrated reductions in hospitalizations and costs, other interventions—such as contextualized clinical decision support or pre-visit contextual data collection—mainly improved communication and care processes without consistently translating into measurable patient outcomes [24–26].

### Reflections on the practice of contextualizing care

While Binns-Calvey et al. (2020) mainly identified contextual red flags based on biomedical or behavioral indicators – such as missed appointments or poor disease control – the specialists in our study tended to recognize red flags earlier in the encounter, often through verbal cues [29]. This finding highlights a more proactive, communication-based approach to identifying contextual factors, indicating that participants in our study were attuned to subtle patient signals rather than relying solely on biomedical cues. However, participants referenced only five of twelve domains from Weiner’s contextual care model [5]. Further research should explore why the remaining domains were absent, including the role of implicit bias. The domains ‘meaning’ and ‘sexuality’ were added to the model during the coding process. While these could be integrated into broader categories like attitude towards illness, their absence in the model reflects a tendency to overlook these factors, resulting in incomplete assessments and care [30–32].

The biggest communication challenge identified was adequately probing contextual red flags. This aligns with research showing that communication training often prioritizes clinical knowledge over nuanced skills [20,27,28]. Gaps in eliciting and interpreting patient context are associated with lower diagnostic accuracy, poorer outcomes, and barriers to SDM or ACP [5,33,34]. Implicit bias may further shape how providers recognize and address contextual factors, contributing to care disparities [35–37]. Tailored, ongoing training is essential, as novice and expert clinicians differ in their ability to navigate patient context [38,39]. Beyond traditional communication training, workplace-integrated interventions like system-level interventions, peer supervision and coaching on the job have shown promising results [40]. This may also help address a challenge mentioned by participants: that other HCPs may recognize or value contextual care to a lesser extent. In particular, multidisciplinary team training could mitigate this by strengthening knowledge, skills, and collaboration both within and across professional groups.

This study shows that medical specialists value contextualized care despite their challenges in integrating it systematically. This does not objectively show though, that patient context *is* indeed impacting treatment plans and outcomes. Using targeted intervention studies, future research should demonstrate a) whether patient context measurably impacts treatment choices, health care outcomes and costs, b) what currently limits the uptake of patient context in care planning, c) which type of interventions most effectively reach optimal integration of patient context into care planning and d) how contextualized care evolves and is maintained over time and across specialties, particularly in multidisciplinary treatment trajectories where information about patient context needs to be transferred between professionals.

### Strengths and limitations

This study’s strengths include a diverse sample of participants across experience levels, ages, and specialties, providing broad insights into contextualized care. Using real-world cases, it highlights how medical specialists interpret and apply patient context. However, several methodological limitations exist. Narrated case histories may omit details, making it hard to track missed context without a standardized system, likely underestimating omissions. Our modified 4C analysis lacked medical record integration, potentially limiting contextual depth. Selection bias is also possible, as participants were already engaged with the topic, excluding perspectives of those who deem patient context less relevant. Additionally, cases may reflect personal selection and recall bias. Future studies could address these limitations.

## Conclusions

This study highlights the critical role of patient context in creating personalized treatment plans that enhance the quality and appropriateness of care. Participants recognized that relevant contextual factors strongly influence clinical decision-making and should be assessed not only at the start of treatment but continuously throughout the treatment process to ensure adaptive and effective care. While all participants acknowledged the value of contextual communication, many struggled to distinguish patient preferences from patient context, which may contribute to contextual errors. Identifying and effectively addressing contextual red flags proved challenging, indicating room for improvement. To better incorporate patient context into healthcare, we recommend: (1) improving communication training to include contextual probing and reflection; (2) encouraging teamwork and consistent sharing of contextual information across specialties; and (3) adding simple contextual prompts or fields in electronic health records to support continuity. Together, these steps can help specialists to connect clinical reasoning with patient’s life circumstances and deliver more person-centered care.

## Supporting information

S1 File COREQ checklist. (PDF)

S2 File The interview guide. (PDF)

S3 File An overview of identified 4C elements in the narrated cases. (PDF)
