# Supplementary material for: “On paper it was a surgical success, but the reality was very different”: A qualitative interview study using narrated casuistry to explore patient context in specialist care
Source: PLoS One. 2026 Jan 12;21(1):e0339353. doi: 10.1371/journal.pone.0339353 (PMC12795376; doi:10.1371/journal.pone.0339353)
Supplement: S2 File — (PDF) [file pone.0339353.s002.pdf]

Supporting Information File 2: The interview guide

1. **What comes to mind from your medical specialty when it comes to contextualizing care? In your opinion, what is or is not the significance of it?**

    - What, in your view, is contextualized care? What does it look like within your specialty?
    - Which contextual factors do you consider from your medical specialty?
    - How do you address contextual factors in treatment?

2. **Could you describe a case or cases where patient context proved to be important?**

    - Which contextual factors were involved? How did you identify them?
    - What made you include or exclude these contextual factors in the treatment? And what did the integration of contextual factors into this treatment look like?
    - How did these contextual factors influence the treatment?

3. **Could you describe a case or cases where patient context was (consciously or unconsciously) not taken into account? Or where you only became aware of it afterwards?**

    - Which contextual factors were involved? Were you aware of these factors? If not, why not?
    - What made you exclude these contextual factors from the treatment?
    - How did these contextual factors influence the treatment?
