# Supplementary material for: “On paper it was a surgical success, but the reality was very different”: A qualitative interview study using narrated casuistry to explore patient context in specialist care
Source: PLoS One. 2026 Jan 12;21(1):e0339353. doi: 10.1371/journal.pone.0339353 (PMC12795376; doi:10.1371/journal.pone.0339353)
Supplement: S3 File — (PDF) [file pone.0339353.s003.pdf]

		Integrated context (N)	Missed context (N)
**Contextual red flag**	**How was the red flag recognized?**	Verbal communication (18)<br>Non-verbal communication (3)<br>Medical facts (11)	Verbal communication (4)<br>Non-verbal communication (3)<br>Medical facts (4)
	**What was recognized/ missed?**	**Verbal communication:**<br>Meaning (2)<br>Treatment preferences (1)<br>Resistance to treatment options (8)<br>Conflicting priorities (2)<br>Hobbies (1)<br>Illness interpretation (1)<br>Repeated statements (1)<br><br>**Non-verbal communication:**<br>Emotion (3)<br>Lifestyle choices (2)<br>Socioeconomic position (1)<br>Capabilities (3)<br><br>**Medical facts:**<br>Unsuccessful treatment (3)<br>Physical status patient (3)<br>Severity of the medical condition (1)<br>Recurrent hospitalization (2)<br>Typical/difficult behaviour (2)<br>Unexplained symptoms (1)	**Verbal communication:**<br>Resistance to treatment options (2)<br>Treatment preferences (1)<br>Social situation (1)<br><br>**Non-verbal communication:**<br>Emotion (3)<br>Capabilities (3)<br><br>**Medical facts:**<br>Incomplete anamnesis (3)<br>Change of care situation (1)<br>Medication usage (1)<br>Physical status patient (1)<br>Late/no-show at appointments (1)
**Contextual probe**	**Why was the red flag probed?**	Potential implications for treatment choice (5)<br>Understanding reason behind:<br>- unsuccessful treatment (1)<br>- resistance to treatment options (4)<br>- recurrent hospitalizations (1)<br>- behaviour (1)<br>- repeated statement (1)<br>- symptoms (1)<br>Exploring patient's preferences regarding treatment plan[1] (5)<br>Minimizing patient's vulnerabilities (1)	
	**How was the red flag probed?**	Medical specialist in same conversation (9)<br>Medical specialist in a later conversation (2)<br>Medical specialist in a repeating conversation (1)	

[1] Patients' preferences were explored but not (necessarily) the reason or meaning behind it.

		Structured exploration medical specialist (1)<br>House visit by medical specialist + general practitioner (1)<br>Delegate to general practitioner (1)<br>Call and ask GP (1)<br>Medical specialist with patient's care givers (1)	
**Contextual domain**	**Which domain was identified/ overlooked?**	Environment (6)<br>Meaning (3)<br>Skills, abilities, knowledge (6)<br>Emotional state (9)<br>Sexuality (1)<br>Attitude towards illness (1)Health behaviour (6)	Emotional state (1)<br>Social support (2)<br>Environment (1)<br>Skills, abilities and knowledge (4)<br>Attitude towards illness (2)
**Contextual plan**	**How was/could the plan (be) contextualized?**	Refrained from treatment (4)<br>Adjusted treatment plan (10)<br>Same treatment and extra support for contextual factor (2)<br>Facilitated right care/support after treatment (4)	Discuss treatment scenarios (3)<br>Facilitate extra support (1)<br><br>**Consequences:**<br>- Patient adapted care to loved ones' wishes<br>- Treatment against patient's preference<br>- Complications after surgery<br>- Psychiatric admission

Supporting Information File 3: An overview of identified 4C elements in the narrated cases
